# Typification of names of South American taxa related to *Woodsia montevidensis* (Woodsiaceae)

**DOI:** 10.3897/phytokeys.63.8366

**Published:** 2016-04-26

**Authors:** Marcelo D. Arana, Claudine M. Mynssen, Brigitte Zimmer, M. Monica Ponce

**Affiliations:** 1 Orientación Botánica II, Departamento de Ciencias Naturales, Facultad de Ciencias Exactas, Físico-Químicas y Naturales, Universidad Nacional de Río Cuarto, Ruta 36 km 601, X5804ZAB Río Cuarto, Córdoba, Argentina; 2 Instituto de Pesquisa Jardim Botânico do Rio de Janeiro, Pacheco Leão 915, 22460-030, Rio de Janeiro, RJ, Brazil; 3 Botanical Garden & Botanical Museum Berlin-Dahlem, Königin-Luise Str. 6-8, D-14195 Berlin, Germany; 4 Instituto de Botánica Darwinion (ANCEFN-CONICET), Labardén 200, Casilla de Correo 22, B1642HYD San Isidro, Buenos Aires, Argentina

**Keywords:** Cheilanthes, Diacalpe, Physematium, nomenclature, Woodsia, Woodsiaceae

## Abstract

A revision of the nomenclature of six South American taxa related to *Woodsia* is presented, as a part of a taxonomic revision of the genus in South America. Lectotypes are selected for *Cheilanthes crenata*, Woodsia crenata var. pallidipes, *Woodsia incisa*, Woodsia montevidensis var. fuscipes and the second step lectotypification for *Dicksonia montevidensis* and *Woodsia peruviana*, based on the analysis of their protologues and original herbarium material. All names are currently synonyms of *Woodsia montevidensis*. *Physematium incisum* (Gillies ex Hook. & Grev.) Kunze constitutes an illegitimate name and *Physematium cumingianum* is considered as nomen inquirendum.

## Introduction

*Woodsia* (Polypodiidae: Woodsiaceae) is a genus comprising about 35-40 taxa of small and tufted ferns growing on or in the vicinity of cliffs and rocks (Brown 1964). They mainly occur in montane areas in the Northern Hemisphere, but few species are also present in South America and Southern Africa. The greatest species richness is found in the Rocky Mountains of western North America (ca. 10 spp.) and the Himalayas in south Asia (ca. 19 spp.); absent from Australia, New Zealand, SE Asia, and the Pacific (Kramer 1990, Shao et al. 2015, Shmakov 2015). The Neotropical species belong to Woodsia subgenus Physematium (Kaulf.) Hook. emend. X.C. Zhang & R. Wei (Shao et al. 2015, Shmakov 2015). South American floristic works cite the genus *Woodsia* as being represented by a single, morphologically variable species, *Woodsia montevidensis* (Spreng.) Hieron., with a distribution ranging from Venezuela and Colombia to central Argentina and Southern Brazil (de la Sota 1977, Tryon and Stolze 1991).

In anticipation of the recent efforts to prepare floristic inventories for Neotropical and Andean regions of South America, especially the Flora of Argentina (http://www.floraargentina.edu.ar) and the Flora of Brazil (Mynssen 2016), the nomenclature of taxa related to the genus *Woodsia* from South America was re-examined, and we are here providing lectotypes for four names, and two second step lectotypifications with the aim of enhancing nomenclatural stability, following as closely as possible the authors’ original intentions.

## Material and methods

We have analysed the protologues and morphological features from specimens of the following herbaria: B, BA, BAB, BM, CONC, CORD, CTES, HB, JUA, K, L, LE, LIL, LP, LZ, MCNS, MERL, MO, MVFA, NY, OXF, P, PR, PRC, R, RB, RCVC, RIOC, S, SI, US and W (acronyms see Thiers 2016). Typification was done according to the current edition of the International Code of Nomenclature for algae, fungi and plants (ICN) (McNeill et al. 2012) and considering the proposal concerning inadvertent lectotypifications and neotypifications (Prado et al. 2015).

## Typifications

In this paper we have arranged the South American taxa related to *Woodsia* in alphabetical order by the names under *Woodsia*, as all of them are current synonyms of *Woodsia montevidensis* (Spreng.) Hieron., following Brown (1964), de la Sota (1977), and Tryon and Stolze (1991).

1. ***Woodsia crenata*** (Kunze) Hieron. Bot. Jahrb. Syst. 34(4): 440. 1904. ≡ *Cheilanthes crenata* Kunze, Linnaea 9: 84. 1834. Type: Peru. “Peruv.(ia) Rupestribus ad Huanuco (6,200’) Martio 1830 lectae” *E.F. Poeppig, s.n.* (Lectotype, designated here: W [W-0061329!]).

Since Kunze’s own herbarium in Leipzig is destroyed, we looked for further original material of *Cheilanthes crenata* from Huanuco, Peru collected by Poeppig at B, BM, K, L, LE, MO, NY, OXF, P, PCR, US and W. We were able to find original material at W that agrees well with Kunze`s original description, which we here select as lectotype, in order to avoid the misapplication of the name.

2. ***Woodsia crenata*** var. ***pallidipes*** Hieron., Bot. Jahrb. Syst. 34(4): 440. 1904. Type: Colombia. “Ad muros et rupes prope Puracé”, 2680-2800 m, 1 Feb 1884, *F.C. Lehmann 3478*. Lectotype (designated here): B [B-200170834!]; isolectotypes: B [B-200170833!], K [K-000632733!], US [US-00066996!].

When Hieronymus (1904) described Woodsia crenata var. pallidipes, he cited four collections, three from Colombia and two from Bolivia. Bolivia: “sine loco, 1863”, *Mandon 19* B [B-200171567!, on the right side of the sheet] and *Mandon 35* B [B-200171567!, on the left side of the sheet]. Colombia: “ad muros urbis Pasto”, 2500 m, 11 Feb 1881, *F.C. Lehmann 656* B [B-20170836!], “ad muros et rupes prope Purace”, 2680-2800 m., 1 Feb 1884, *F.C. Lehmann 3478* B [B-20170834!, B-2017083!], K [K-000632733!], US [US-00066996!], “ad muros et rupes prope Yermal, in provincia Antioquia”, 1800-2400 m, Nov 1891, *F.C. Lehmann 7411* B [B-20170835!], K [K-000632732!]. We selected a specimen from the *F.C. Lehmann 3478* collection as lectotype because it corresponds with all characters used to describe the variety, furthermore the B specimen has a handwritten label by Hieronymus with the inscription “Woodsia crenata var. pallidipes Hieron.” and there are duplicates in three herbaria.

3. ***Woodsia incisa*** Gillies ex Hook. & Grev., Icon. Filic. 2. t. 191. 1831 ≡ *Physematium incisum* (Gillies ex Hook. & Grev.) C. Presl, Tent.: 66. 1836. Type: Argentina. Mendoza: near San Luis, *J. Gillies s.n.* Lectotype (designated here): BM [BM-000937851!]; isolectotypes BM [BM-000937850!]; K [K-000229420!].

The type material at BM consists of four fronds with two different barcodes on the same sheet: BM [BM-000937850 and BM-000937851], both with separate labels with the same information. We selected the material affiliated with BM [BM-000937851] as lectotype because it is more complete.

The specimen *J. Gillies 8* housed at K [K-000229420!] is not part of the original material because it was collected at “Sierras de Tandil”, located in Buenos Aires province, Argentina, far away from the type locality.

The combination *Physematium incisum* (Gillies ex Hook. & Grev.) Kunze (Kunze 1837) is an illegitimate name, posterior to Presl’s combination.

4. ***Woodsia montevidensis*** (Spreng.) Hieron., Bot. Jahrb. Syst. 22: 363. 1896. ≡ *Dicksonia montevidensis* Spreng., Syst. Veg. 4(1): 122. 1827. Type: Uruguay. (“Brasilia”) [Montevideo], Pan d’Açucar, *F. Sellow d 517.* Lectotype (first step designated by Tryon & Stolze [1991: 94]), second step (designated here): B [B-200094654!]; isolectotype B [B-200120343!].

The protologue only expresses “Monte Video. *Sello*”. There are seven specimens of Sellow from Montevideo, five of them are kept in B, one in BM and another in K. Tryon and Stolze (1991: 94) typified *Woodsia montevidensis* with a specimen at B. From all specimens deposited in B, two of them are numbered *Sellow d 517* B [B-200094654! and B-200120343!] from Montevideo, as well quoted by Hieronymus when he made the combination under *Woodsia* (Hieronymus 1896). The specimen B [B-200094654!] is selected here as lectotype because it corresponds with all characters used to describe the species, and probably it was the specimen seen by Sprengel because it bears the annotation of G. Hieronymus “Original von Sprengel”. Also it shows on a second label n. 118. “(Sprengel)” on a third: “Pan d’Açucar”, and on the fourth: “d.517”. The specimen B [B-200120343!] is considered isolectotype.

The remaining specimens: B [B-200170837a], Montevideo, ex reliquiis Sellowianis, s.n., ded. Humboldt 1836, ex herb. Kunth, [the two fronds on the left], B [B-200170837b], Montevideo, Pan d’ Açucar, ex reliquiis Sellowianis, s.n., ded. Humboldt 1836 [the two fronds on the right], B [B-200120342 and B-200120344] bear the only annotation “Brasilia” without specific locality (same label Herb. Reg. Berolinense, as K [K-000632729!], and BM [BM-000937849!], although probably being original material, are preferably excluded from lectotypification because the data of the label are not complete.

5. ***Woodsia montevidensis*** var. ***fuscipes*** Hieron., Hedwigia 46: 322. 1907. Type: Argentina. Salta. “Prov. de Salta, Los Potreros al pie del Nevado del Castillo, 24.03.1827”, *P.G. Lorentz & G.H.E.W. Hieronymus 138.* Lectotype (designated here): B [B-200171577!]; isolectotypes: B [B-200171580!, B-200171581!], CORD!.

When Hieronymus described the variety *fuscipes*, he mentioned five collections in the protologue. Two collections from Bolivia: Illimani between Pongo and Apachate. alt.: 4350 m, 24 March 1873, collected by *A. Stübel 1239* (B [B-200171573!]) and La Paz, Murillo, Zongo (“prope Songo”), Nov 1890, collected by *M. Bang 878* (B [B-200171572!], MO [MO-1919967 digital image!]; P [P-01400358!]; PH [PH-00029464 digital image!]; UC not seen; US [US-00067000!]). From Argentina, three additional collections were considered by Hieronymus as belonging to this variety: *F. Schickendantz* 68 (B [B-200171578!, B-200171579!]), *F. Schickendantz* 360 (B [B-200171576!]), and *P.G. Lorentz & G.H.E.W. Hieronymus 138* (B [B-200171577!, B-200171580!, B-200171581!], CORD!). Specimens of all five collections are present at B. In order to avoid any ambiguity regarding the application of the name, the specimen *Lorentz & Hieronymus 138* (B [B-200171577!]) is selected as lectotype, while the three duplicates are regarded as isolectotypes in accordance to Art. 9.12 of the Code (McNeill et al. 2012). Also, the lectotype chosen shows the characters used to delimitate the variety and bears an annotation by Hieronymus “n. var,” and handwritten locality data.

6. ***Woodsia peruviana*** Hook., Sp. Fil. 1: 61, pl. 21B. 1844. ≡ *Diacalpe peruviana* (Hook.) Trevis., Nuovo Giorn. Bot. Ital.7: 160. 1875. Type: Peru. “Huamantanga, shady places”, 1834-1835, *A. Mathews 602*. Lectotype (first step designated by Tryon & Stolze (1991: 94), second step (designated here): K [K-000632731!]; isolectotypes B [B-20 0094655!, B-20 0171563!], BM [BM-000937848 digital image!], GH [GH-00022287 digital image!], and K [K-000632730 digital image!].

Tryon and Stolze (1991: 94) typified *Woodsia peruviana* with a specimen at K as holotype, but K holds two sheets of *Mathews 602* [K-000632730 and K-000632731], the last one is here designated as lectotype because the material is more complete, has a handwritten annotation “Peru, Mathews” and “*Woodsia peruviana* Hook. Spec. Fil. Tab. XXI” on the sheet, and the label contains the locality data.

The specimen *A. Mathews s.n.* (US [US-00067001!]), according with Taylor’s annotation in the label of the specimen, could probably be part of the type collection, but we prefer to exclude it of lectotypification because the locality is not clear (only “Peru” is written in the label) and it is not originally numbered by Mathews.

## Unresolved name

*Woodsia cumingiana* (Kunze) Hook., Sp. Fil. [W. J. Hooker] 1: 61. 1844. ≡ *Physematium cumingianum* Kunze, Analecta Pteridogr.: 43. 1837. Type: “Habitat probabiliter in Chile, misit *H. Cuming*” (Herb. Kunze in LZ, destroyed).

The original material of this species, deposited in LZ, was destroyed. As Kunze (1837) observed in the protologue: “unicum vidi specimen observed”, there is not referable isotype or even an illustration of the species. According to Stafleu and Cowan (1979), the original material of H. Cuming is kept at BM; however, no syntypes were found in this herbarium, nor in the Herbarium Hookerianum (K). Additionally, no material of this species from Chile was in B, BR, BM, E, GH, L, LE, OXF, P, W and Z, where duplicates of H. Cuming are deposited. Also, as consigned by Hooker (1844), most probably the type locality is mistaken, because he had the opportunity to revise the collections of Cuming immediately after his return and he was not able not find any specimen gathered by Cuming from either Chile or Peru. As the protologue expresses, the species is characterized by last segments oblong rounded, glanduloso-dentate decurrent, sori solitary upon the teeth, involucres glabrous, rachis and stipe subglabrous purple. With such description, the species is hardly to differenciate from many species of *Woodsia*, hence, the name is considered here as nomen inquirendum.
